# The *BRCA1 *Ashkenazi founder mutations occur on common haplotypes and are not highly correlated with anonymous single nucleotide polymorphisms likely to be used in genome-wide case-control association studies

**DOI:** 10.1186/1471-2156-8-68

**Published:** 2007-10-04

**Authors:** Lutécia H Mateus Pereira, Marbin A Pineda, William H Rowe, Libia R Fonseca, Mark H Greene, Kenneth Offit, Nathan A Ellis, Jinghui Zhang, Andrew Collins, Jeffery P Struewing

**Affiliations:** 1 Laboratory of Population Genetics, National Cancer Institute, Bethesda, MD 20892, USA; 2 Department of Biological Sciences, Florida International University, University Park, Miami, FL 33199, USA; 3 Clinical Genetics Branch, National Cancer Institute, Bethesda, MD 20892, USA; 4 Memorial Sloan Kettering Cancer Center, Clinical Genetics Service, Department of Medicine, New York, NY 10021, USA; 5 Gastroenterology Section, Department of Medicine, University of Chicago, Chicago, IL 60637, USA; 6 Human Genetics Division, University of Southampton, Southampton General Hospital, Southampton SO16 6YD, UK

## Abstract

**Background:**

We studied linkage disequilibrium (LD) patterns at the *BRCA1 *locus, a susceptibility gene for breast and ovarian cancer, using a dense set of 114 single nucleotide polymorphisms in 5 population groups. We focused on Ashkenazi Jews in whom there are known founder mutations, to address the question of whether we would have been able to identify the 185delAG mutation in a case-control association study (should one have been done) using anonymous genetic markers. This mutation is present in approximately 1% of the general Ashkenazi population and 4% of Ashkenazi breast cancer cases. We evaluated LD using pairwise and haplotype-based methods, and assessed correlation of SNPs with the founder mutations using Pearson's correlation coefficient.

**Results:**

*BRCA1 *is characterized by very high linkage disequilibrium in all populations spanning several hundred kilobases. Overall, haplotype blocks and pair-wise LD bins were highly correlated, with lower LD in African *versus *non-African populations. The 185delAG and 5382insC founder mutations occur on the two most common haplotypes among Ashkenazim. Because these mutations are rare, even though they are in strong LD with many other SNPs in the region as measured by D-prime, there were no strong associations when assessed by Pearson's correlation coefficient, *r *(maximum of 0.04 for the 185delAG).

**Conclusion:**

Since the required sample size is related to the inverse of *r*, this suggests that it would have been difficult to map *BRCA1 *in an Ashkenazi case-unrelated control association study using anonymous markers that were linked to the founder mutations.

## Background

Numerous advances in our understanding of genetic susceptibility to breast cancer have been made over the past decade, most notably the discovery of *BRCA1 *in 1994 and *BRCA2 *in 1995 [1,2]. Mutations in these genes account for approximately 2/3 of families with clearly inherited forms of breast and ovarian cancer (5 or more cases in a family) [3,4]. In addition to the high-penetrance genes *BRCA1/BRCA2*, rare mutations in a number of other genes, such as *CHEK2*, *ATM*, *BRIP1*, and *PALB1 *predispose to breast cancer [5-9], as do more common variants in *CASP8 *and *TGFB1*[10]. The total heritability of breast cancer is at least 10% [11,12], and possibly up to 25% or higher [13,14]. Mutations in known high-risk genes, however, account for a relatively small proportion (probably less than 20%) of the excess risk due to genetic factors [15,16].

Fueled by the completion of the first phases of the HapMap project [17], which characterized common variation within the genome of four population groups, there is considerable interest in using these resources to map susceptibility genes for common, complex diseases. The genome-wide case-control association study, whereby the prevalence of genetic variants is compared between cases and unrelated control subjects without the disease, may have the greatest power to identify novel susceptibility genes [18-20]. They rely on using a very dense set of markers that capture a significant fraction of all common genetic variation, such that the variants assayed either include those that are biologically relevant, or those which are highly-correlated with the former due to linkage disequilibrium. Although some design and analysis issues remain, numerous common variants have now been identified for breast cancer and other conditions using this design [21,22].

Breast cancer may serve as a useful paradigm for common, complex disease mapping studies because, while a portion of susceptibility genes have been identified, the majority of the residual familial clustering remains unexplained, and is likely to be polygenic in nature, due to a number of lower-penetrance genes in the context of environmental exposures [23,24]. Furthermore, there are two common Ashkenazi Jewish (AJ) founder *BRCA1 *mutations, 185delAG and 5382insC, initially identified in linkage studies of multiple-case breast/ovarian cancer families [25]. This contrasts with most other populations in which there are numerous unique *BRCA1/BRCA2 *mutations, with none present at 1% or greater population frequency. The *BRCA1 *AJ founder mutations account for the majority of Jewish breast-ovarian cancer families, and are present in approximately 1% of the general Jewish population [26]. The AJ founder mutations, owing to their high prevalence compared to other populations, offered an opportunity to test whether they might have been identified through a case-control association study of the kind suggested as the new gene discovery strategy in the post-HapMap era.

## Results

### SNP allele frequencies

A total of 289 unrelated reference subjects selected without regard to breast cancer from five population groups (48 each from African-Americans, Chinese-Americans and Mexican-Americans, 60 CEPH subjects, and 85 Ashkenazi Jews) were genotyped across *BRCA1*, spanning a region of approximately 646 kb. Table 1 presents the allele frequencies and Hardy-Weinberg *P*-vales for all the 112 polymorphic SNPs and the two founder mutations that were typed for all 5 populations. Eight of 570 tests showed departures from equilibrium at the 0.01 level, but because none of the eight showed Mendelian segregation errors within families, and because of the number of comparisons performed, they were not excluded from later calculations. Allele frequencies were generally highly correlated among Ashkenazi Jews, CEPH, Chinese-Americans and Mexican-Americans (minimum *r *>0.82), whereas African-Americans presented the lowest correlation values with all the other populations (maximum *r *<0.44). The highest correlation was found between Ashkenazi Jews and CEPH (*r *= 0.947) and the lowest between African-Americans and Mexican-Americans (*r *= 0.362). Most of the private SNPs (*n *= 18) originated in the African-American samples, although private SNPs were also observed in Ashkenazi Jews (*n *= 4), Chinese-Americans (*n *= 3), and Mexican-Americans (*n *= 1) (Table 1). Total observed heterozygosity for each marker across the five populations ranged from 0.4% for private SNPs to 49.3% [see Additional file 1]. F_ST _ranged from 0.0047 (SNP8 – rs8176072) to 0.4338 (SNP102 – rs2593595). Sixty three percent of the SNPs showed little genetic differentiation (F_ST _< 0.05), followed by twenty eight percent with moderate (0.05–0.15) and less than ten percent with higher genetic differentiation. We also calculated pair-wise F_ST _measures, and the distribution was very similar for the Ashkenazi Jews, CEPH, Chinese-Americans and Mexican-Americans *versus *each other (range from 0.008–0.018) as compared with African-Americans *versus *all the other populations (range from 0.082–0.092), showing that African-Americans had by far the greatest level of differentiation. These results are congruent with the low allele frequency correlation values observed between African-Americans *versus *all the other groups.

**Table 1:** SNP Frequencies and Hardy-Weinberg P-Values

							Ashkenazi Jews (n = 85)		CEPH (n = 60)		African Americans (n = 48)		Chinese Americans (n = 48)		Mexican Americans (n = 48)	
rs number	SNP Number	SNP name	Position b33^a^	Position b36.1^b^	Com Allele^c^	Min Allele^d^	maf^e^	*P*-value	maf	*P*-value	maf	*P*-value	maf	*P*-value	maf	*P*-value
13119	1	C_9270420	41373486	38718247	c	t	0.424	0.580	0.350	0.712	0.177	0.624	0.396	0.359	0.458	0.961
748620	2	C_9270421	41373037	38717798	c	g	0.440	0.640	0.350	0.712	0.240	0.550	0.391	0.518	0.465	0.669
17599948	3	C_9270454	41364175	38708936	a	g	0.211	0.649	0.192	0.865	0.104	0.022	0.094	0.326	0.362	0.591
11653231	4	C_95201	41240225	38584986	g	a	0.435	0.703	0.350	0.712	0.240	0.550	0.396	0.359	0.467	0.905
9908805	5	C_3178692	41230675	38575436	c	t			0.008	0.948	0.375	0.878			0.011	0.941
2175957	6	C_11631183	41195587	38540348	t	g	0.447	0.665	0.350	0.712	0.250	0.441	0.396	0.359	0.446	0.935
3092986	7	BR1_000340	41186761	38531522	a	g	0.051	0.636	0.042	0.736	0.010	0.942			0.031	0.823
8176072	8	BR1_000392	41186709	38531470	t	a	0.006^f^	0.957								
8176074	9	BR1_000571	41186530	38531291	g	a					0.010	0.942				
3765640	10	C_2615164	41185012	38529773	a	g	0.440	0.640	0.350	0.712	0.228	0.740	0.394	0.433	0.444	0.592
NA^g^	11	M_185delAG	41184811	38529572	a	-	0.006	0.957								
8176090	12	BR1_007918	41179183	38523944	c	g			0.008	0.948	0.096	0.338				
1800062	13	BR1_010574	41176528	38521289	g	a							0.021	0.883		
8176101	14	BR1_010796	41176306	38521067	a	t					0.021	0.882				
8176103	15	C_2615171	41175815	38520576	g	a	0.463	0.291	0.350	0.712	0.234	0.640	0.396	0.359	0.458	0.961
8176104	16	BR1_011340	41175762	38520523	g	a	0.024	0.824	0.033	0.789					0.010	0.942
8176109	17	C_2615172	41174541	38519302	a	g	0.441	0.497	0.350	0.712	0.239	0.609	0.396	0.359	0.469	0.751
8065872	18	BR1_016775	41170328	38515089	t	a					0.135	0.278			0.010	0.942
8176120	19	BR1_017105	41169998	38514759	g	a	0.424	0.913	0.350	0.712	0.240	0.550	0.396	0.359	0.458	0.961
799914	20	BR1_018573	41168546	38513307	g	a					0.135	0.278				
799913	21	BR1_019408	41167711	38512472	a	g	0.032	0.772	0.042	0.736	0.365	0.813			0.010	0.942
8176128	22	BR1_019904	41167215	38511976	t	a					0.063	0.644				
8176133	23	BR1_020896	41166223	38510984	t	g	0.422	0.738	0.350	0.712	0.167	0.488	0.396	0.359	0.458	0.961
799912	24	C_2615180	41165899	38510660	c	t	0.472	0.701	0.364	0.926	0.125	0.322	0.396	0.359	0.489	0.882
799923	25	BR1_026422	41160696	38505457	c	t	0.232	0.031	0.308	0.858	0.042	0.763			0.104	0.459
8176145	26	BR1_029258	41157859	38502620	t	c	0.400	0.038	0.319	0.586	0.240	0.550	0.404	0.309	0.448	0.829
8176146	27	BR1_029448	41157669	38502430	c	t	0.031	0.774	0.008	0.948						
7503154	28	BR1_030748	41156369	38501130	t	g	0.415	0.618	0.339	0.478	0.223	0.771	0.396	0.359	0.468	0.862
1799950	29	BR1_031875	41155246	38500007	a	g	0.077	0.442	0.042	0.736	0.010	0.942			0.031	0.823
4986850	30	BR1_032885	41154236	38498997	g	a	0.133	0.096	0.113	0.071	0.033	0.819			0.013	0.935
16940	31	BR1_033119	41154002	38498763	t	c	0.422	0.429	0.350	0.712	0.177	0.624	0.396	0.359	0.448	0.829
799917	32	BR1_033420	41153701	38498462	c	t	0.429	0.766	0.358	0.868	0.125	0.322	0.396	0.359	0.479	0.571
4986852	33	BR1_003927	41153194	38497955	g	a	0.012	0.912	0.042	0.736	0.010	0.942				
2227945	34	BR1_034226	41152895	38497656	a	g					0.052	0.703			0.010	0.942
16942	35	BR1_034356	41152765	38497526	a	g	0.433	0.868	0.348	0.615	0.245	0.521	0.396	0.359	0.447	0.716
799916	36	C_7530109	41151955	38496716	t	g	0.438	0.482	0.358	0.868	0.271	0.703	0.394	0.433	0.479	0.894
2070833	37	BR1_035507	41151614	38496375	c	a	0.031	0.001					0.271	0.726	0.135	0.167
2070834	38	BR1_036077	41151050	38495811	a	c	0.435	0.626	0.342	0.568	0.250	1.000	0.396	0.359	0.438	0.634
8176158	39	BR1_036793	41150334	38495095	a	g	0.418	0.393	0.353	0.665	0.174	0.532	0.396	0.359	0.458	0.961
8176160	40	BR1_036859	41150268	38495029	a	g	0.435	0.626	0.350	0.712	0.240	0.550	0.396	0.359	0.458	0.961
8176166	41	BR1_038085	41149042	38493803	a	g	0.196	0.391	0.150	0.172	0.104	0.022	0.120	0.632	0.344	0.395
8176174	42	BR1_040350	41146777	38491538	a	t					0.063	0.644				
3950989	43	C_3178696	41146718	38491479	g	a	0.441	0.566	0.348	0.615	0.234	0.640	0.396	0.359	0.457	0.923
8176175	44	BR1_040669	41146458	38491220	t	-	0.006	0.956					0.073	0.586		
8176177	45	BR1_041288	41145840	38490601	a	g					0.021	0.883				
8176178	46	BR1_041721	41145407	38490168	a	g					0.042	0.763				
1060915	47	C_3178676	41143235	38487996	a	g	0.380	0.105	0.352	0.851	0.177	0.624	0.396	0.359	0.468	0.862
3737559	48	C_3178677	41143069	38487830	c	t	0.124	0.481	0.067	0.580			0.083	0.208	0.043	0.761
8176187	49	BR1_045154	41141974	38486735	t	c									0.010	0.942
8176188	50	BR1_045505	41141623	38486384	t	g					0.063	0.644				
6416927	51	BR1_0046019	41141109	38485870	g	c							0.083	0.208		
8176198	52	BR1_047826	41139302	38484063	t	a	0.422	0.429	0.362	0.427	0.348	0.114	0.426	0.761	0.479	0.571
8176199	53	BR1_0477839	41139289	38484050	a	c	0.341	0.594	0.258	0.502	0.135	0.167	0.177	0.624	0.396	0.135
4239147	54	BR1_048551	41138577	38483338	t	c	0.440	0.640	0.324	0.677	0.271	0.703	0.394	0.433	0.452	0.711
8176206	55	BR1_050244	41136885	38481646	a	g					0.052	0.703			0.010	0.942
2236762	56	C_11621042	41135440	38480201	a	t	0.435	0.626	0.358	0.868	0.271	0.703	0.406	0.581	0.479	0.894
1799966	57	C_2615208	41131859	38476620	t	c	0.456	0.456	0.350	0.712	0.233	0.781	0.396	0.359	0.469	0.751
3092987	58	BR1_055669	41131488	38476249	a	g	0.416	0.545	0.350	0.712	0.170	0.510	0.396	0.359	0.458	0.961
8176225	59	BR1_056796	41130361	38475122	g	t					0.031	0.823				
8176232	60	BR1_058369	41128788	38473549	c	t	0.013	0.936								
8176234	61	BR1_058614	41128545	38473306	a	g	0.437	0.345	0.350	0.712	0.239	0.609	0.396	0.359	0.458	0.961
8176235	62	BR1_058834	41128325	38473086	g	a	0.339	0.872	0.258	0.502	0.167	0.488	0.396	0.359	0.436	0.972
8176236	63	BR1_059589	41127570	38472331	t	c			0.008	0.948	0.359	0.556			0.010	0.942
8176240	64	BR1_060022	41127137	38471898	t	c					0.063	0.644				
8176242	65	BR1_060520	41126639	38471400	g	a	0.433	0.538	0.350	0.712	0.170	0.510	0.406	0.581	0.458	0.961
8176245	66	BR1_061014	41126145	38470906	t	c					0.063	0.644				
3092994	67	C_2615220	41124590	38469351	c	t	0.434	0.471	0.350	0.712	0.228	0.242	0.396	0.359	0.266	0.808
8176259	68	BR1_062588	41124571	38469332	t	-					0.052	0.703				
8176265	69	BR1_0064398	41122761	38467522	g	a	0.417	0.477	0.350	0.712	0.167	0.729	0.394	0.433	0.260	0.848
2187603	70	BR1_064501	41122658	38467419	g	a	0.422	0.429	0.350	0.712	0.167	0.729	0.396	0.359	0.260	0.848
8176273	71	BR1_066741	41120418	38465179	t	c	0.424	0.580	0.342	0.568	0.167	0.729	0.396	0.359	0.260	0.848
8176278	72	BR1_067978	41119181	38463942	a	g			0.008	0.948	0.500	0.564			0.021	0.883
8066171	73	BR1_068063	41119096	38463857	g	t					0.146	0.237			0.010	0.942
NA	74	M_5382insC	41117845	38462606	-	c	0.006	0.957								
8176289	75	C_2615230	41114821	38459582	t	c	0.441	0.566	0.342	0.568	0.223	0.258	0.396	0.359	0.266	0.808
8176293	76	BR1_073023	41114136	38458897	a	-							0.031	0.823		
4793192	77	BR1_074008	41113155	38457916	a	g	0.435	0.626	0.336	0.794	0.229	0.214	0.396	0.359	0.260	0.848
8176296	78	BR1_074807	41112356	38457117	a	g	0.435	0.703	0.350	0.712	0.229	0.214	0.396	0.359	0.260	0.848
3092988	79	C_2615238	41110467	38455228	c	t	0.424	0.580	0.350	0.712	0.170	0.709	0.396	0.359	0.260	0.848
8176303	80	BR1_076933	41110230	38454991	a	g					0.010	0.942	0.042	0.763		
8176305	81	BR1_077034	41110129	38454890	a	g	0.094	0.753	0.092	0.442	0.021	0.883			0.010	0.942
8176307	82	BR1_077328	41109835	38454596	t	c					0.063	0.644				
8068463	83	BR1_079220	41107943	38452704	c	t					0.125	0.322				
8176313	84	BR1_079396	41107767	38452528	g	a	0.006	0.957	0.017	0.896						
8176316	85	BR1_080570	41106594	38451355	c	a					0.021	0.883				
8176318	86	BR1_081125	41106039	38450800	g	t	0.435	0.963	0.350	0.712	0.167	0.729	0.385	0.491	0.250	1.000
12516	87	BR1_081990	41105173	38449934	c	t	0.435	0.626	0.350	0.712	0.229	0.214	0.406	0.581	0.260	0.848
8176320	88	BR1_082035	41105128	38449889	g	a	0.006	0.957	0.033	0.789					0.010	0.942
8176321	89	BR1_082199	41104964	38449725	g	a					0.010	0.942				
8176323	90	BR1_082687	41104476	38449237	c	g	0.429	0.766	0.350	0.712	0.229	0.214	0.396	0.359	0.260	0.848
7223952	91	C_11621012	41103649	38448411	t	c	0.441	0.566	0.356	0.765	0.281	0.569	0.396	0.359	0.287	0.931
9911630	92	C_3178665	41097108	38441868	a	g	0.422	0.429	0.364	0.926	0.266	0.808	0.396	0.359	0.283	0.813
11460963	93	C_2615245	41090062	38435121	-	g	0.441	0.497	0.350	0.712	0.228	0.242	0.396	0.359	0.266	0.808
2298861	94	C_3178699	41085596	38430357	g	a	0.441	0.309	0.350	0.712	0.223	0.258	0.396	0.359	0.266	0.808
2298862	95	C_3178698	41085453	38430214	t	c	0.447	0.383	0.350	0.712	0.208	0.343	0.396	0.359	0.260	0.848
443759	96	C_2287905	41074499	38419260	c	t	0.229	0.129	0.241	0.656	0.354	0.520	0.083	0.208	0.146	0.981
11871636	97	C_11617231	41063582	38408343	a	c	0.235	0.304	0.259	0.386	0.302	0.345	0.167	0.083	0.135	0.278
2271539	98	C_1588447	41059714	38404475	a	g	0.388	0.932	0.388	0.339	0.213	0.447	0.448	0.341	0.277	0.768
690971	99	C_765227	41025330	38370091	g	t					0.281	0.885				
528854	100	C_1588417	41006873	38351634	a	g	0.106	0.230	0.098	0.488	0.479	0.990			0.063	0.644
323495	101	C_1588405	40983252	38328013	g	a	0.316	0.182	0.342	0.568	0.208	0.001	0.396	0.135	0.443	0.407
2593595	102	C_3256885	40965010	38309771	a	g	0.124	0.007	0.054	0.672	0.156	0.002	0.135	0.883	0.177	<.0001
324075	103	C_3256881	40935288	38280049	a	g	0.171	0.717	0.195	0.063	0.115	0.370	0.156	0.365	0.330	0.558
2290041	104	C_15883310	40856085	38200846	c	t	0.012	0.913			0.302	0.103	0.031	0.823		
1078523	105	C_2160077	40821525	38166286	a	g	0.480	0.158	0.370	0.650	0.135	0.883	0.478	0.369	0.474	0.075
752313	106	C_1075621	40810589	38155350	t	c	0.363	0.314	0.422	0.851	0.292	0.954	0.406	0.250	0.351	0.251
7359598	107	C_3256867	40806234	38150996	c	t	0.386	0.073	0.440	0.674	0.135	0.883	0.438	0.486	0.383	0.243
2271027	108	C_15959277	40779567	38124328	c	t					0.065	0.636	0.031	0.823		
7214055	109	C_1441435	40761936	38106697	c	g	0.149	0.109	0.008	0.948	0.385	0.003	0.031	0.823	0.021	0.882
9766	110	C_1441436	40761606	38106367	g	a	0.353	0.219	0.433	0.700	0.292	0.954	0.406	0.250	0.344	0.823
1553469	111	C_7529639	40751527	38096288	a	c	0.065	0.248	0.058	0.061	0.031	<.0001			0.202	0.943
2271029	112	C_1125369	40744687	38089448	c	a	0.405	0.009	0.350	0.444	0.375	0.441	0.385	0.937	0.402	0.117
3760384	113	C_1441438	40744216	38088977	a	c	0.359	0.020	0.392	0.666	0.447	0.716	0.385	0.937	0.458	0.023
2292749	114	C_1441444	40727349	38072110	c	t	0.217	0.219	0.383	0.518	0.192	0.794	0.375	0.878	0.128	0.316

Number of polymorphic loci							83		80		99		71		82	
Number of SNPs with maf<0.05							11		13		14		6		17	
Mean maf							0.305		0.269		0.181		0.327		0.287	

^a ^April 2003 (Build 33) Position

^b ^March 2006 (Build 36.1) Position

^c ^Com Allele = Common allele

^d ^Min Allele = Minor allele

^e ^maf = Minor allele frequency

^f ^Population private SNPs are shown underlined

^g^NA= Not Applicable – not in dbSNP

### LD structure

In order to analyze the LD structure at the *BRCA1 *locus, we chose two methods that rely on different premises. The first is *haplotype block analysis *which identifies sequential and non-overlapping sets of variants in high LD, separated by low levels of LD that are consistent with historical recombination. In this method, all htSNPs need to be genotyped in order to capture most of the genetic variation [27]. The second is a *binning method *in which SNPs in one LD bin can be interleaved with SNPs in other overlapping bins. Under this approach, one TagSNP per bin needs to be tested in order to capture SNP diversity [28].

**Figure 1:**
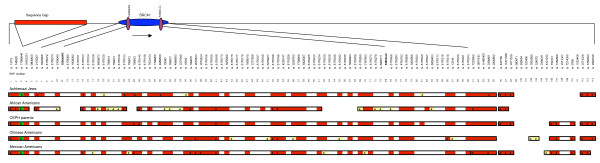
**Comparison of haplotype blocks at 114 loci across five populations**. Blocks were defined as in [27]; markers with MAF <0.05 are shown with a white background and were ignored in the calculations and block boundary estimation. Haplotype tag SNPs (htSNPs) within a block are indicated by arrowheads; htSNPs in only one population are shown on a yellow background while the single htSNP shared between all populations is shown on a green background.

**Figure 2:**
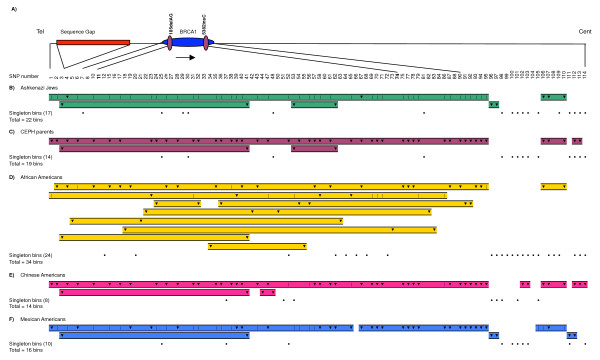
**Comparison of SNP bins derived from pair-wise measurements of linkage disequilibrium using LDSelect-Comp**. SNPs with MAF < 5% do not have a vertical line or arrowhead in the column. A) Scale representation of the ~650 kb region studied, indicating the *BRCA1 *gene, founder mutations, and genome sequence gap of unknown true size. Anchor lines link to position of the SNP within the region. B-F) LDSelect creates bins of SNPs that have an *r*^2 ^value of 0.8 or greater with at least one other SNP in the bin. Each vertical line and arrowhead represents a SNP, with dashed lines and shaded background connecting SNPs within the same bin. Down arrowheads indicate Tag SNPs (those with *r*^2 ^≥ 0.8 with all other SNPs in a bin). Note that this use of the term Tag-SNP is different from Haploview – with LDSelect, only one Tag-SNP per bin would be required to capture the majority of the nucleotide diversity. Singleton bins (SNPs that did not have *r*^2 ^≥ 0.8 with any other SNP) are indicated by solid dots on a single row. SNP number refers to numbering in column 1 of Table 1.

**Figure 3:**
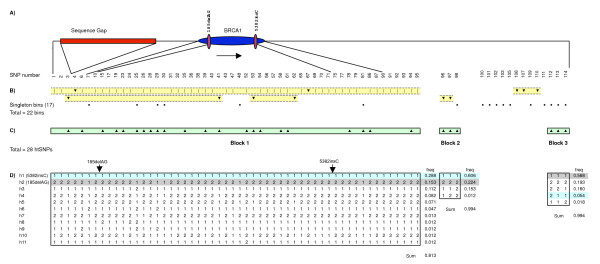
**Pair-wise measures of linkage disequilibrium and the two founder mutation-containing haplotypes**. SNP number refers to numbering in column 1 of Table 1; only the 70 with MAF ≥ 0.05 in Ashkenazi Jews are shown in B-D. A) Scale representation of the ~650 kb region studied, indicating the *BRCA1 *gene, founder mutations, and genome sequence gap of unknown true size. B) LDSelect-Comp output showing a total of 22 bins for Ashkenazi Jews, with 17 "singleton" bins indicated by solid dots on a single row. C) Haploview output showing three block structures and related ht-SNPs (indicated with up arrowheads). D) Haplotypes estimated for 85 unrelated Ashkenazi Jews using SNPHAP as implemented in HapScope. The block boundaries were calculated in Haploview and overlaid on this figure. All haplotypes with an estimated frequency of at least 1% are displayed (h1 to h11), with individual frequencies and sums indicated to the right of the blocks. The common allele is designated "1" and the minor allele "2". The 185delAG and 5382insC containing haplotypes, determined from the family based genotypes, are indicated with gray (haplotype 2) and blue background (haplotype 1), respectively. Black arrows indicate the relative position of these two founder mutations.

Our analyses of D' and *r*^2 ^showed *BRCA1 *residing in a large region (~288 kb) of high LD (Fig. 1), in agreement with other reports [29-31]. The entire region studied showed long-range LD, falling primarily into three blocks among non-African populations. The block containing *BRCA1 *includes 95 SNPs and overlaps the largest LDSelect bin of SNPs correlated at *r*^2 ^> 0.8 (Fig. 2 and Fig. 3).

African-Americans presented the least LD of all populations, with the presence of more distinct blocks within the region (Fig. 1). Maps for all five populations shared a break-point that maps approximately 20 kb downstream of the *BRCA1 *gene, between SNP95 (rs2298862) and SNP96 (rs443759). Among non-African groups, only Mexican-Americans exhibited an additional break point within the 288 kb block structure that encompasses *BRCA1*. The 3'end of the entire region showed less extensive LD but a similar pattern across all the groups. Only one htSNP (SNP3 – rs17599948) was found to be completely shared across populations, which is not unexpected since htSNPs often are population-specific [28] (Fig. 1).

When the bin-based approach was used, we found that bins were largely shared across different ethnic groups (Fig. 2). The differences across populations were related to the number of bins as well as the number and position of TagSNPs. As expected, African-Americans were the most diverse group, containing the highest number of bins (34), followed by Ashkenazi Jews (22), CEPH (19), Mexican-Americans and Chinese-American (14). This contrasts with 28 htSNPs in African-Americans, and 16, 13, 25 and 18 among Ashkenazi Jews, CEPH, Mexican- Americans and Chinese-Americans, respectively. Three TagSNPs were shared by all populations (SNP3 – rs17599948, SNP41 – rs8176166, and SNP67 – rs3092994), showing average MAF of 0.193, 0.183 and 0.335, respectively (Fig. 2). Mexican-Americans showed two disjoint bins of highly-correlated SNPs, rather than one extended bin structure as evidenced in Ashkenazim, CEPH and Chinese-Americans. The disjoint occurred between positions 38,471,400 (SNP65 – rs8176242) and 38,469,351 (SNP67 – rs3092994), mapping between introns 17 and 18 of *BRCA1 *(Fig. 2F). Interestingly, our results resemble what others have observed [32] in Native- Americans, namely an historical recombination event between introns 15 and 18. All five populations showed a large bin spanning ~288 kb encompassing SNP1 (rs13119) through SNP95 (rs2298862) (Fig. 2A–F), which represented the same extended region found in the block analysis. This large bin had 0.278 average MAF across populations and included *BRCA1 *coding polymorphisms L771L_(TTG>CTG), P871L_(CCG>CTG), 1183R_(AAA>AGA) and S1436S_(TCT>TCC) (Fig. 2).

**Figure 4:**
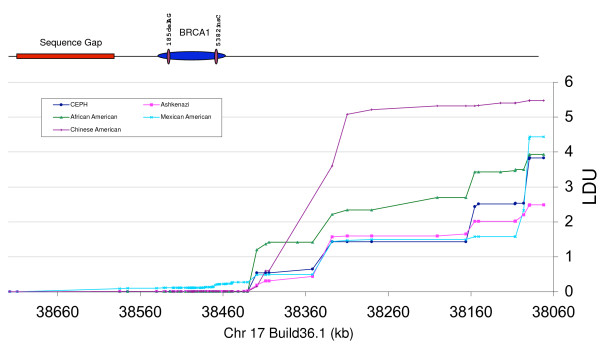
**LDU maps. Comparison of LDU maps across the ~650 kb region containing the *BRCA1 *gene for 5 populations**. Top. Scale representation of the ~650 kb region studied, indicating the *BRCA1 *gene, founder mutations, and genome sequence gap of unknown true size. Bottom. LDU maps for five populations.

The maps of linkage disequilibrium in LD units (LDU) corresponded well with the two previous approaches of assessing disequilibrium. The four major breakpoints that were observed in Fig. 1 and 2, when haplotype blocks and bin structures were inferred, coincided with the same major steps in the LDU analysis (Fig. 4). In addition, we were able to observe two small steps in Fig. 4 for the Mexican-Americans, which were not observed in any other population. The first step occurred between SNP65 (rs8176242, intron 17) and SNP69 (rs8176265, intron 19). The corresponding site of possible recombination could be observed as a split at the main bin structure for Mexican-Americans in Fig. 2F. The second step was found between SNP90 (rs8176323) and SNP91 (rs7223952), downstream of the gene. A close correspondence was evidenced as a breakdown in LD around the same position in Fig. 1.

### 185delAG and 5382insC haplotype reconstruction

We were particularly interested in fine mapping the *BRCA1 *locus to identify possible gene variants or haplotypes associated with the two founder mutations in Ashkenazi Jews. Therefore, 82 intragenic and 30 flanking SNPs were tested against the two founder mutations with the Pearson's correlation (*r*) coefficient (Table 2). Although in strong LD with the majority of markers as measured by D' (Table 2), the highest pair-wise *r*^2 ^for 185delAG was 0.04, owing to its relatively low frequency, with a common SNP (SNP96, rs443759) that mapped outside the large *BRCA1*-containing block, approximately 110 kb downstream of the gene. Regarding 5382insC, there was one highly-significant association (*r*^2 ^= 1.0) with SNP8 (rs8176072) (Table 2). This is a rare SNP that was present only in 5382insC mutation carriers, and 5382insC was not correlated above 0.03 with any other SNP in the region.

**Table 2:** Pair-wise correlation coefficients between the two founder *BRCA1 *mutations and all other SNPs among Ashkenazi Jews

				Ashkenazim (n = 85)			Correlation (*r *^2^) with		D' with	D' with
					
SNP name	rs number	SNP number	SNP description	maf^a^	Het^b^	HW *P*-value	185delAG	5382insC	185delAG	5382insC
C_9270420	13119	1	NBR1: UTR	0.424	0.52	0.580	0.011	0.001	1.000	1.000
C_9270421	748620	2	NBR1: UTR	0.440	0.52	0.640	0.016	0.000	1.000	1.000
C_9270454	17599948	3	NBR1: intron	0.211	0.35	0.649	0.016	0.001	1.000	1.000
C_95201	11653231	4	NBR1: intron	0.435	0.51	0.703	0.014	0.001	1.000	1.000
C_3178692	9908805	5	LBRCA1: UTR	0.000					1.000	1.000
C_11631183	2175957	6	NBR2: intron	0.447	0.52	0.665	0.013	0.001	1.000	1.000
BR1_000340	3092986	7	NBR2: intron	0.051	0.10	0.636	0.001	0.000	1.000	1.000
BR1_000392	8176072	8	NBR2:intron	0.006^c^	0.01	0.957	0.000	1.000	1.000	1.000
BR1_000571	8176074	9	NBR2:UTR	0.000					1.000	1.000
C_2615164	3765640	10	BRCA1:UTR	0.440	0.52	0.640	0.015	0.000	1.000	1.000
M_185delAG	NA^d^	11	BRCA1:exon 2	0.006	0.01	0.957	NA	0.000	NA	1.000
BR1_007918	8176090	12	BRCA1: intron	0.000					1.000	1.000
BR1_010574	1800062	13	BRCA1: P_K38K_(AAG>AAA)	0.000					1.000	1.000
BR1_010796	8176101	14	BRCA1: intron	0.000					1.000	1.000
C_2615171	8176103	15	BRCA1: intron	0.463	0.56	0.291	0.014	0.000	1.000	1.000
BR1_011340	8176104	16	BRCA1: intron	0.024	0.05	0.824	0.001	0.000	1.000	1.000
C_2615172	8176109	17	BRCA1: intron	0.441	0.53	0.497	0.011	0.000	1.000	0.000
BR1_016775	8065872	18	BRCA1: intron	0.000					1.000	1.000
BR1_017105	8176120	19	BRCA1: intron	0.424	0.49	0.913	0.015	0.001	1.000	1.000
BR1_018573	799914	20	BRCA1: intron	0.000					1.000	1.000
BR1_019408	799913	21	BRCA1: intron	0.032	0.06	0.772	0.001	0.000	1.000	1.000
BR1_019904	8176128	22	BRCA1: intron	0.000					1.000	1.000
BR1_020896	8176133	23	BRCA1: intron	0.422	0.51	0.738	0.012	0.000	1.000	1.000
C_2615180	799912	24	BRCA1: intron	0.472	0.52	0.701	0.011	0.000	1.000	0.000
BR1_026422	799923	25	BRCA1: intron	0.232	0.44	0.031	0.003	0.000	1.000	1.000
BR1_029258	8176145	26	BRCA1: intron	0.400	0.59	0.038	0.021	0.001	1.000	0.000
BR1_029448	8176146	27	BRCA1: intron	0.031	0.06	0.774	0.001	0.000	1.000	1.000
BR1_030748	7503154	28	BRCA1: intron	0.415	0.51	0.618	0.012	0.001	1.000	1.000
BR1_031875	1799950	29	BRCA1: P_Q356R_(CAG>CGG)	0.077	0.15	0.442	0.002	0.000	1.000	1.000
BR1_032885	4986850	30	BRCA1: P_D693N_(GAC>AAC)	0.133	0.19	0.096	0.001	0.000	0.000	1.000
BR1_033119	16940	31	BRCA1: P_L771L_(TTG>CTG)	0.422	0.53	0.429	0.012	0.001	1.000	1.000
BR1_033420	799917	32	BRCA1:P_P871L_(CCG>CTG)	0.429	0.51	0.766	0.015	0.001	1.000	1.000
BR1_003927	4986852	33	BRCA1: P_S1040N_(AGC>AAC)	0.012	0.02	0.912	0.000	0.000	1.000	1.000
BR1_034226	2227945	34	BRCA1: P_S1140G_(AGT>GGT)	0.000					1.000	1.000
BR1_034356	16942	35	BRCA1: P_K1183R_(AAA>AGA)	0.433	0.50	0.868	0.015	0.001	1.000	1.000
C_7530109	799916	36	BRCA1: intron	0.438	0.53	0.482	0.015	0.000	1.000	1.000
BR1_035507	2070833	37	BRCA1: intron	0.031	0.04	0.001	0.001	0.000	1.000	1.000
BR1_036077	2070834	38	BRCA1: intron	0.435	0.52	0.626	0.014	0.001	1.000	1.000
BR1_036793	8176158	39	BRCA1: intron	0.418	0.54	0.393	0.012	0.000	1.000	1.000
BR1_036859	8176160	40	BRCA1: intron	0.435	0.52	0.626	0.014	0.001	1.000	1.000
BR1_038085	8176166	41	BRCA1: intron	0.196	0.35	0.391	0.018	0.000	0.388	1.000
BR1_040350	8176174	42	BRCA1: intron	0.000					1.000	1.000
C_3178696	3950989	43	BRCA1: intron	0.441	0.52	0.566	0.007	0.000	1.000	1.000
BR1_040669	8176175	44	BRCA1: intron	0.006	0.01	0.956	0.000	0.000	1.000	1.000
BR1_041288	8176177	45	BRCA1: intron	0.000					1.000	1.000
BR1_041721	8176178	46	BRCA1: intron	0.000					1.000	1.000
C_3178676	1060915	47	BRCA1: P_S1436S_(TCT>TCC)	0.380	0.56	0.105	0.018	0.001	1.000	0.000
C_3178677	3737559	48	BRCA1: intron	0.124	0.20	0.481	0.001	0.001	0.462	1.000
BR1_045154	8176187	49	BRCA1: intron	0.000					1.000	1.000
BR1_045505	8176188	50	BRCA1: intron	0.000					1.000	1.000
BR1_0046019	6416927	51	BRCA1: intron	0.000					1.000	1.000
BR1_047826	8176198	52	BRCA1: intron	0.422	0.53	0.429	0.016	0.001	1.000	1.000
BR1_0477839	8176199	53	BRCA1: intron	0.341	0.42	0.594	0.016	0.000	1.000	1.000
BR1_048551	4239147	54	BRCA1: intron	0.440	0.52	0.640	0.011	0.001	1.000	1.000
BR1_050244	8176206	55	BRCA1: intron	0.000					1.000	1.000
C_11621042	2236762	56	BRCA1: intron	0.435	0.52	0.626	0.016	0.001	1.000	1.000
C_2615208	1799966	57	BRCA1: intron	0.456	0.54	0.456	0.008	0.000	1.000	0.185
BR1_055669	3092987	58	BRCA1: intron	0.416	0.52	0.545	0.013	0.002	1.000	1.000
BR1_056796	8176225	59	BRCA1: intron	0.000					1.000	1.000
BR1_058369	8176232	60	BRCA1: intron	0.013	0.03	0.936	0.000	0.000	1.000	1.000
BR1_058614	8176234	61	BRCA1: intron	0.437	0.54	0.345	0.016	0.000	1.000	1.000
BR1_058834	8176235	62	BRCA1: intron	0.339	0.44	0.872	0.017	0.000	1.000	1.000
BR1_059589	8176236	63	BRCA1: intron	0.000					1.000	1.000
BR1_060022	8176240	64	BRCA1: intron	0.000					1.000	1.000
BR1_060520	8176242	65	BRCA1: intron	0.433	0.52	0.538	0.011	0.001	1.000	1.000
BR1_061014	8176245	66	BRCA1: intron	0.000					1.000	1.000
C_2615220	3092994	67	BRCA1: intron	0.434	0.53	0.471	0.012	0.000	1.000	0.000
BR1_062588	8176259	68	BRCA1: intron	0.000					1.000	1.000
BR1_0064398	8176265	69	BRCA1: intron	0.417	0.52	0.477	0.012	0.001	1.000	1.000
BR1_064501	2187603	70	BRCA1: intron	0.422	0.53	0.429	0.009	0.001	1.000	1.000
BR1_066741	8176273	71	BRCA1: intron	0.424	0.52	0.580	0.011	0.001	1.000	1.000
BR1_067978	8176278	72	BRCA1: intron	0.000					1.000	1.000
BR1_068063	8066171	73	BRCA1: intron	0.000					1.000	1.000
M_5382insC	NA	74	BRCA1:exon 20	0.006	0.01	0.957	0.000	NA	1.000	NA
C_2615230	8176289	75	BRCA1: intron	0.441	0.52	0.566	0.014	0.000	1.000	1.000
BR1_073023	8176293	76	BRCA1: intron	0.000					1.000	1.000
BR1_074008	4793192	77	BRCA1: intron	0.435	0.52	0.626	0.016	0.001	1.000	1.000
BR1_074807	8176296	78	BRCA1: intron	0.435	0.51	0.703	0.015	0.000	1.000	1.000
C_2615238	3092988	79	BRCA1: intron	0.424	0.52	0.580	0.015	0.001	1.000	1.000
BR1_076933	8176303	80	BRCA1: intron	0.000					1.000	1.000
BR1_077034	8176305	81	BRCA1: intron	0.094	0.16	0.753	0.002	0.001	1.000	1.000
BR1_077328	8176307	82	BRCA1: intron	0.000					1.000	1.000
BR1_079220	8068463	83	BRCA1: intron	0.000					1.000	1.000
BR1_079396	8176313	84	BRCA1: intron	0.006	0.01	0.957	0.000	0.000	1.000	1.000
BR1_080570	8176316	85	BRCA1: intron	0.000					1.000	1.000
BR1_081125	8176318	86	BRCA1: UTR	0.435	0.49	0.963	0.009	0.001	1.000	1.000
BR1_081990	12516	87	BRCA1: UTR	0.435	0.52	0.626	0.014	0.001	1.000	1.000
BR1_082035	8176320	88	BRCA1: UTR	0.006	0.01	0.957	0.000	0.000	1.000	1.000
BR1_082199	8176321	89	Intergenic	0.000					1.000	1.000
BR1_082687	8176323	90	Intergenic	0.429	0.51	0.766	0.015	0.001	1.000	1.000
C_11621012	7223952	91	Intergenic	0.441	0.52	0.566	0.014	0.000	1.000	1.000
C_3178665	9911630	92	Intergenic	0.422	0.53	0.429	0.016	0.001	1.000	1.000
C_2615245	11460963	93	Intergenic	0.441	0.53	0.497	0.014	0.001	1.000	1.000
C_3178699	2298861	94	ARHN: locus	0.441	0.55	0.309	0.011	0.001	0.000	1.000
C_3178698	2298862	95	ARHN: locus	0.447	0.54	0.383	0.011	0.001	1.000	1.000
C_2287905	443759	96	IFI35:intron	0.229	0.41	0.129	0.040	0.002	1.000	1.000
C_11617231	11871636	97	RPL27: intron	0.235	0.40	0.304	0.036	0.002	1.000	1.000
C_1588447	2271539	98	RPL27: intron	0.388	0.47	0.932	0.023	0.003	1.000	1.000
C_765227	690971	99	MGC2744: intron	0.000					1.000	1.000
C_1588417	528854	100	MGC2744: intergenic	0.106	0.16	0.230	0.000	0.001	1.000	1.000
C_1588405	323495	101	G6PC: intergenic	0.316	0.37	0.182	0.004	0.000	0.205	1.000
C_3256885	2593595	102	G6PC: intron	0.124	0.15	0.007	0.000	0.001	1.000	1.000
C_3256881	324075	103	Intergenic	0.171	0.29	0.717	0.000	0.000	0.266	1.000
C_15883310	2290041	104	PRKWNK4: mis-sense	0.012	0.02	0.913	0.005	0.000	1.000	1.000
C_2160077	4321242	105	RAMP2: intron	0.480	0.58	0.158	0.003	0.000	1.000	1.000
C_1075621	752313	106	EZH1: intergenic	0.363	0.40	0.314	0.005	0.000	1.000	1.000
C_3256867	7359598	107	EZH1: intergenic	0.386	0.57	0.073	0.005	0.001	1.000	0.000
C_15959277	2271027	108	EZH1: intron	0.000					1.000	1.000
C_1441435	7214055	109	CNTNAP1: UTR	0.149	0.30	0.109	0.001	0.001	1.000	1.000
C_1441436	9766	110	CNTNAP1: UTR	0.353	0.52	0.219	0.007	0.000	0.173	1.000
C_7529639	1553469	111	CNTNAP1: silent	0.065	0.11	0.248	0.002	0.000	0.463	1.000
C_1125369	2271029	112	CNTNAP1: silent	0.405	0.62	0.009	0.000	0.001	0.040	1.000
C_1441438	3760384	113	CNTNAP1: intron	0.359	0.58	0.020	0.005	0.000	1.000	1.000
C_1441444	2292749	114	TUBG2: intron	0.217	0.39	0.219	0.006	0.001	1.000	1.000

^a ^maf = Minor allele frequency

^b^Het = Heterozygosity

^c^Population private SNPs are shown underlined

^d^NA= Not Applicable

Haplotypes were estimated for the set of all SNPs with MAF ≥ 0.05. Haplotypes for the founder mutation containing chromosomes were unambiguously determined in at least one family across the entire region studied. The185delAG and 5382insC mutations occurred on the two most common haplotypes, representing 15% (haplotype 2) and 29% (haplotype 1) of the chromosomes, respectively, among Ashkenazi Jews (Fig. 3D). In the haplotype analyses, the 185delAG mutation occurred on a chromosome with the minor allele at most loci, and the 5382insC on a chromosome with the major allele at most loci (Fig. 3D). This pattern constitutes what has been previously described as "yin yang haplotypes", in which two high-frequency haplotypes have different alleles at most SNP sites [33].

## Discussion

The primary objective of this study was to address the question of whether we could identify the Ashkenazi *BRCA1 *founder mutation 185delAG in a typical case-control association study, using anonymous genetic markers. The answer is no. The impact on the required sample size (S) needed if one studies a marker in LD with the true disease allele is related to the inverse of the square of their correlation coefficient (*r*), as in S = 1/*r*^2 ^[34]. Almost none of the SNPs had a high correlation coefficient with either founder mutation (Table 2). Since most markers were more common than the founder mutations, this result is not surprising. However, our SNP selection strategy did not exclude low frequency SNPs. In fact, one of the SNPs identified in 3 of the 90 Polymorphism Discovery Resource subjects [35] was perfectly correlated with 5382insC. However, we did not observe this SNP in any of the four non-Ashkenazi reference populations. Based on our results, the sample size required to detect the 185delAG mutation in a breast cancer case-control study conducted in Ashkenazi women that did not directly test for the mutation, would be at least 25 times larger than one that measured the mutation directly, requiring on the order of 62,000 subjects. Based on pair-wise measurements, we conclude that it would have been extremely difficult to have mapped the two founder mutations using the case-control association methodology using common SNPs.

Association studies may also compare combinations of SNPs, or haplotypes, between cases and controls, and the founder mutations might have been discoverable if they occurred on uncommon haplotypes. Using relatively common SNPs (MAF ≥ 5%), like those on whole-genome SNP platforms, we found that the two mutations were present on haplotypes representing a polar pattern, termed yin-yang haplotypes [33]. These two haplotypes accounted together for a large percentage of the total chromosomes studied, independent of the population, ranging from 64% for the Chinese-Americans to 43% for the CEPH.

It is highly unlikely that the founder mutations could have been discovered owing to a difference in haplotype frequency between cases and controls largely because they occur on the two most common haplotypes. For example, consider a case-control study of Ashkenazi Jews with 500 cases and 500 controls. Among controls (1000 chromosomes), the distribution of *BRCA1*-containing haplotypes would be roughly as in Fig. 3D (i.e., there would have been 288 chromosomes with haplotype 1 and 153 with haplotype 2). Among cases, assuming 1% carried 5382insC and 4% carried 185delAG, there would be 5 additional haplotype 1 (total = 293) and 20 additional haplotype 2 (total = 173) chromosomes. These case-control contrasts, 293 vs. 288 (OR 1.02) and 173 vs. 153, would require extremely large sample sizes of over 30,000 subjects to detect either mutation with 80% statistical power. Conversely, in the more advantageous situation in which the 185delAG mutation by chance occurred on a rare haplotype (for example, haplotype 8), there would have been 32 such chromosomes in cases vs. 12 in controls requiring approximately 4,800 subjects for the same statistical power.

The *BRCA1 *locus is well known to have significant LD [29,30]. Nonetheless, we found a marked differentiation between African-Americans and non-African Americans in the haplotype block analysis. Compared with African-Americans, the non-African American populations had less haplotype diversity and more extensive LD (Fig. 1). The increased number of crossovers along the entire region for African-Americans probably reflects older evolutionary events. Our data conform to previous findings [27], describing higher haplotype diversity as well as less extensive LD in the Yoruban and African American samples than in European and Asian populations. When SNP "bins" derived from pair-wise measurements of LD were compared, we found a greater extent of LD boundaries being shared across the five different ethnical groups (Fig. 2). Ashkenazi Jews and the CEPH population had highly similar patterns of LD, independently of the type of analysis used to generate the LD structures (haplotype, or pair-wise bin methods) (Fig. 1 and Fig. 2). Overall, haplotype blocks and bins showed similar patterns, probably owing to the strong LD present overall in this region. The LDU analysis showed a remarkable overall similarity with the two previous methods that were used to analyze LD (Fig. 4). There were basically four major breakdowns in LD downstream to *BRCA1 *that were largely shared across populations. Nevertheless, African-Americans presented more recombination events than the other four populations, consistent with the smaller block sizes showed in Fig. 1.

## Conclusion

In summary, our detailed analyses of 114 polymorphic SNPs in a 646 kb region around *BRCA1 *in Ashkenazi Jews and other populations confirmed a high level of linkage disequilibrium across nearly the entire region. In addition to 85 unrelated Ashkenazi Jews, we over-sampled carriers of the founder mutations 185delAG and 5382insC and their relatives to more precisely calculate correlations with other markers and to molecularly determine the mutation associated haplotypes (these subjects were not included in allele frequency estimates). This allowed us to assess the likelihood of discovering the founder mutations by virtue of their association with individual SNPs or haplotypes that one would assay in a breast cancer case-control study in Ashkenazi Jews. We did not observe a high correlation coefficient between any individual SNP likely to be included in a genome-wide anonymous scan and either founder mutation. Our findings suggest that a study at least 25X larger (60,000 subjects or more) would have been required if the mutations were not tested for directly. The two founder mutations occur on the two most common haplotypes, representing over 40% of the chromosomes, also suggesting that a haplotype-based analysis would not have been successful at detecting either of the underlying mutations. These results are influenced heavily by the relative rarity of the founder mutations, as reflected by high values for Lewontin's D' measures of LD but low correlation coefficients. Our results suggest caution in using genome-wide association studies with common SNPs for detecting uncommon, disease-causing mutations.

## Methods

### Subjects

Independent subjects included 85 unrelated Ashkenazi Jews, 60 European-Americans (Utah) from the CEPH (The Centre d'Etude du Polymorphisme Humain) family collection, and 48 each from African-Americans, Chinese-Americans and Mexican-Americans (Human Diversity Collection, Coriell Cell Repository, Camden, NJ). The 30 children of the 60 Utah CEPH subjects were also assayed to test for Mendelian errors. In addition, six unrelated *BRCA1*:185delAG and three unrelated *BRCA1*:5382insC founder mutation carriers and their relatives [36], identified through the National Cancer Institute's Cancer Family Registry, were included in the study in order to establish mutation-associated haplotypes from family data. Mutation-associated haplotypes were inferred through inspection of genotypes for all available first-degree relatives of mutation carriers. The Ashkenazi Jewish samples were obtained from anonymous control subjects from the National Laboratory for the Genetics of Israeli Populations at Tel-Aviv University [37].

### Marker selection and genotyping

The 90 kb *BRCA1 *locus was previously re-sequenced in 90 individuals representing five major US ethnic/population groups from the Polymorphism Discovery Resource (PDR-90) [35], by the University of Washington as part of the Environmental Genome Project (EGP) [38]. Samples consisted of 24 European-, 24 African- 24 Asian-, 12 Mexican-, and six Native-Americans. The geographic origin of individual donors, however, is masked and may not be used to assign allele frequencies to specific sub-populations. Most of the 301 variants identified were SNPs. Genotyping all 301 variants at this locus in the current study was not necessary since many are highly correlated. We developed the following strategy to identify a reduced set of variants that still captured much of the diversity of the region. Using the EGP data on all 299 biallelic single nucleotide substitutions (i.e., no lower minor allele frequency cutoff), and using custom software, we calculated all pair-wise correlations (*r*^2^) and created "clusters", defined as groups of SNPs that were perfectly correlated with all others in the cluster. Our method is similar to LDSelect 1.0 [28] except that it required that all pair-wise correlations of SNPs in a cluster be 1.0 (i.e., complete LD). LDSelect is typically used with a threshold value of *r*^2 ^of 0.8, and SNPs are clustered into "LD bins" if their pair-wise *r*^2 ^is at or above this threshold value with at least one other SNP (but not all) in the bin. Using an *r*^2 ^of 1.0 resulted in more clusters than a lower *r*^2 ^threshold, increasing the number of SNPs assayed in this study.

Taqman 5'-nuclease assays were developed through Applied Biosystems (Foster City, CA) Assay-by-Design service after first filtering for repetitive, non-unique, and low-complexity sequence. We developed assays for all "singleton" SNPs (those that did not have pair-wise *r*^2 ^values of 1.0 with another SNP). For the 59 clusters with two or more SNPs, we chose one SNP from each cluster of two, three and four SNPs, and for 9 clusters of five or more SNPs, we chose one fourth of them for assay development. In addition, we selected all (*n *= 43) commercially available Assay-on-Demand assays (Applied Biosystems, Foster City, CA) that mapped within approximately 200 kb upstream and 400 kb downstream of the *BRCA1 *locus. This SNP set represented almost all known variants (or ones highly correlated) at this locus.

Of the 143 resulting assays, three were excluded due to technical problems (poor clustering or more than one Mendelian error), and 28 were not polymorphic in our complete sample set, leaving 112 polymorphic SNPs in addition to the two founder mutations. There were 82 *BRCA1 *intragenic SNPs (approximately one SNP per 1 kb) and 30 SNPs that mapped to the region outside *BRCA1 *(approximately one SNP per 20 kb). The allelic discrimination assays were performed in 5 microliter reactions in 384-well plates according to manufacturer's recommendations. Data were analyzed with the allelic discrimination SDS 2.1 software on an ABI 7900HT (Applied Biosystems, Foster City, CA), with manual determination of genotype clusters [see Additional file 2].

### Statistical analysis

Allelic frequency and chi-square goodness-of-fit tests for Hardy-Weinberg equilibrium (HWE) were calculated using SAS/Genetics 9.1 (SAS Institute, Inc., Cary, North Carolina). To assess the correlation between the two founder mutations and all other SNPs, we over-sampled mutation carriers and calculated a weighted Pearson's correlation coefficient using SAS 9.1. We also tested association by use of Tagger [39] operates in either pairwise or aggressive mode, and we used both approaches to examine association. Heterozygosity levels, as well as the variation in gene frequencies between populations by means of their F_ST _(Wright's *F*-statistics) were calculated using POPGENE 1.31 [40].

Haplotypes and their frequencies were inferred from genotypes across the entire region for each population separately, using the software package SNPHAP 1.3 [41], as implemented in Hapscope [42], for loci with minor allele frequencies (MAF) > 5%. SNPHAP uses the expectation-maximization algorithm to calculate maximum likelihood estimates of haplotype frequencies from unphased genotype data.

In order to compare LD patterns across different populations, we employed two different analyses, the first based on partitioning SNPs into haplotype blocks [27] using Haploview [43] and the second based on "bins" of correlated SNPs not constrained to be adjacent to each other [28]. The binning method used a modified version of LDSelect 1.0 that calculates composite LD measures [44], without assuming that loci are in Hardy-Weinberg equilibrium. We used an *r*^2 ^threshold of 0.8 for binning SNPs, and filtered SNPs with population-specific MAF ≤ 0.05.

LDSelect identifies tagSNPs, representing those SNPs in a bin that have *r*^2 ^values at or above the threshold with all other SNPs in a bin. Only one tagSNP in each bin needs to be assayed to capture the majority of the SNP diversity. The block method employed by Haploview groups adjacent SNPs in strong LD, defined as those with one-sided upper 95% confidence bound on D' >0.98 and the lower bound >0.7. In this method, haplotype tag SNPs (htSNPs) represents the set of SNPs that must be assayed in each block to capture all haplotypes at 1% frequency in the population.

LD maps were constructed from genotype data with the software LDMAP [45]. LD maps are scaled in linkage disequilibrium units (LDU) and show (when plotted against the physical map) a pattern of plateaus (reflecting regions of low haplotype diversity and low recombination) and steps (representing regions of historical recombination events).

We genotyped related individuals from families segregating 185delAG and 5382insC founder mutations in order to reconstruct their haplotypes. The 185delAG- and 5382insC-containing haplotypes were unambiguously determined from analyzing the genotypes of all available family members. The frequencies of these mutation-containing haplotypes were determined from SNPHAP analyses of the five populations separately. Block boundaries were defined based on Haploview analyses and overlaid upon the SNPHAP results.

We estimated the required number of subjects to have 80% statistical power to identify the 185delAG mutation if tested directly in a case-control study to be approximately 2492 using EpiInfo 4.0 [46], assuming equal numbers of cases and controls, alpha of 0.0001, and heterozygous carrier frequencies of 0.6% for controls and 3.3% for cases.

## Competing interests

The author(s) declares that there are no competing interests.

## Authors' contributions

LMP performed laboratory and statistical analysis and drafted the manuscript. MAP and LRF performed laboratory analysis. WHR and JZ wrote custom software and performed statistical analysis. MHG provided biomaterials and critically revised the manuscript. KO and NAE participated in the study design and analysis. AC provided design assistance and performed statistical analysis. JPS conceived and designed the study and performed statistical analysis. All authors read and approved the final manuscript.

## Supplementary Material

Additional file 1 Summary of F-statistics and heterozygosity for all loci. Heterozygosity levels, as well as the variation in gene frequencies between populations by means of their F_ST _(Wright's F-statistics). Click here for file

Additional file 2 Genotypes for all 114 polymorphic SNPs in 5 populations. Raw genotypes. Populations: (1) CEPH (includes related individuals), (2) African Americans, (3) Chinese Americans, (4) Mexican Americans and (5) Ashkenazi Jews. Click here for file
