# Supplementary material for: The BRCA1 Ashkenazi founder mutations occur on common haplotypes and are not highly correlated with anonymous single nucleotide polymorphisms likely to be used in genome-wide case-control association studies
Source: BMC Genet. 2007 Oct 4;8:68. doi: 10.1186/1471-2156-8-68 (PMC2093936; doi:10.1186/1471-2156-8-68)
Supplement: Additional file 1 — Summary of F-statistics and heterozygosity for all loci. Heterozygosity levels, as well as the variation in gene frequencies between populations by means of their FST (Wright's F-statistics). [file 1471-2156-8-68-S1.pdf]

Summary of F-Statistics and heterozygosity for all loci

rs number	SNP number	F_{ST}	Het[a]
13119	1	0.042	0.460
748620	2	0.027	0.471
17599948	3	0.060	0.283
11653231	4	0.027	0.477
9908805	5	0.302	0.083
2175957	6	0.023	0.484
3092986	7	0.014	0.060
8176072	8	0.005	0.004
8176074	9	0.008	0.004
3765640	10	0.027	0.480
NA[b]	11	0.005	0.004
8176090	12	0.069	0.028
1800062	13	0.017	0.007
8176101	14	0.017	0.007
8176103	15	0.030	0.489
8176104	16	0.013	0.031
8176109	17	0.028	0.488
8065872	18	0.100	0.049
8176120	19	0.025	0.474
799914	20	0.111	0.045
799913	21	0.234	0.120
8176128	22	0.051	0.021
8176133	23	0.046	0.452
799912	24	0.135	0.462
799923	25	0.114	0.257
8176145	26	0.024	0.507
8176146	27	0.018	0.021
7503154	28	0.030	0.470
1799950	29	0.023	0.076
4986850	30	0.054	0.102
16940	31	0.040	0.460
799917	32	0.141	0.450
4986852	33	0.019	0.028
2227945	34	0.033	0.021
16942	35	0.023	0.470
799916	36	0.069	0.488
2070833	37	0.137	0.105
2070834	38	0.021	0.467
8176158	39	0.043	0.461
8176160	40	0.026	0.481
8176166	41	0.050	0.283
8176174	42	0.051	0.021
3950989	43	0.027	0.477
8176175	44	0.053	0.028
8176177	45	0.017	0.007
8176178	46	0.034	0.014
1060915	47	0.041	0.475
3737559	48	0.029	0.122
8176187	49	0.008	0.004
8176188	50	0.051	0.021
6416927	51	0.068	0.021
8176198	52	0.041	0.461
8176199	53	0.049	0.336
4239147	54	0.077	0.493
8176206	55	0.033	0.021
2236762	56	0.068	0.483
1799966	57	0.031	0.488
3092987	58	0.044	0.458
8176225	59	0.025	0.010
8176232	60	0.010	0.005
8176234	61	0.026	0.488
8176235	62	0.043	0.415
8176236	63	0.288	0.087

8176240	64	0.051	0.021
8176242	65	0.046	0.456
8176245	66	0.051	0.021
3092994	67	0.027	0.472
8176259	68	0.042	0.017
8176265	69	0.039	0.446
2187603	70	0.041	0.450
8176273	71	0.041	0.443
8176278	72	0.411	0.087
8066171	73	0.109	0.053
NA	74	0.005	0.004
8176289	75	0.029	0.465
8176293	76	0.025	0.010
4793192	77	0.028	0.467
8176296	78	0.028	0.465
3092988	79	0.040	0.448
8176303	80	0.025	0.017
8176305	81	0.040	0.090
8176307	82	0.051	0.021
8068463	83	0.103	0.042
8176313	84	0.009	0.010
8176316	85	0.017	0.007
8176318	86	0.043	0.433
12516	87	0.029	0.464
8176320	88	0.016	0.021
8176321	89	0.008	0.004
8176323	90	0.027	0.464
7223952	91	0.089	0.476
9911630	92	0.097	0.474
11460963	93	0.028	0.472
2298861	94	0.029	0.476
2298862	95	0.034	0.467
443759	96	0.051	0.338
11871636	97	0.022	0.347
2271539	98	0.122	0.456
690971	99	0.238	0.066
528854	100	0.259	0.186
323495	101	0.076	0.356
2593595	102	0.434	0.147
324075	103	0.336	0.310
2290041	104	0.213	0.106
4321242	105	0.095	0.494
752313	106	0.039	0.479
7359598	107	0.131	0.482
2271027	108	0.036	0.032
7214055	109	0.193	0.153
9766	110	0.037	0.490
1553469	111	0.073	0.104
2271029	112	0.052	0.497
3760384	113	0.036	0.476
2292749	114	0.055	0.372

[a]Het= Observed heterozygosity

[b]NA= Not Applicable - not in dbSNP
